# Dapagliflozin Beyond Glucose Lowering: Mechanisms of Renal and Systemic Protection

**DOI:** 10.3390/pathophysiology33030068

**Published:** 2026-09-10

**Authors:** Madison L. Wise, Abdel A. Alli

**Affiliations:** 1 Division of Nephrology, Hypertension, and Renal Transplantation, Department of Medicine, University of Florida College of Medicine, Gainesville, FL 32610, USA; madisonwise@ufl.edu; 2 Department of Physiology and Aging, University of Florida College of Medicine, Gainesville, FL 32610, USA

**Keywords:** sodium glucose cotransporter-2 inhibitor, kidney, type 2 diabetes mellitus, phlorizin, epithelial transport proteins, dapagliflozin

## Abstract

Sodium glucose cotransporter-2 inhibitors (SGLT2is) have rapidly evolved from glucose-lowering agents to multifaceted therapies with significant renoprotective and cardioprotective potential. Although originally developed to inhibit glucose reabsorption within the renal proximal tubule for the treatment of Type 2 diabetes mellitus (T2DM), growing evidence indicates that SGLT2is exert broad systemic actions extending beyond glycemic control. Among this drug class, dapagliflozin has emerged as a clinically important agent with pleiotropic effects involving renal hemodynamics, inflammatory signaling, mitochondrial function, fibrosis regulation, and cellular stress adaptation. This review outlines the historical progression from the discovery of phlorizin to the development of highly selective modern SGLT2 inhibitors while emphasizing mechanistic insights gained from experimental and clinical studies of dapagliflozin. In addition to the established effects on sodium–glucose transport, dapagliflozin modulates multiple epithelial transport proteins including NHE3, NaPi-2a, NCC, and NCX1, highlighting complex regulatory effects on sodium handling and tubular electrolyte transport. Emerging evidence further demonstrates that dapagliflozin suppresses inflammatory and profibrotic pathways involving YAP/TAZ, STAT1, TGF-β, NLRP3, and NF-KB signaling. Restoration of tubuloglomerular feedback, attenuation of oxidative stress, and preservation of mitochondrial function also appear to contribute substantially to the renoprotective actions of SGLT2 inhibition. Beyond the kidney, dapagliflozin and related SGLT2is exert cardioprotective effects through coordinated improvements in cardiac energetics, inflammatory regulation, and hemodynamic function. Emerging studies additionally suggest potential pulmonary benefits, including reductions in inflammatory signaling, pulmonary edema, and respiratory complications. Collectively, these findings support a shift in understanding SGLT2is from targeted metabolic therapies to broader regulators of cellular and organ function. Continued investigation into the glucose-independent mechanisms of dapagliflozin may reveal additional therapeutic applications across chronic metabolic, cardiovascular, and inflammatory diseases.

## 1. Introduction

Diabetes Mellitus is a set of diseases categorized by high blood sugar, or hyperglycemia. Hyperglycemia is a result of ineffective insulin secretion, insulin activity, or a combination of the two [1]. Unfortunately, diabetes has become a major disease affecting millions of people globally. As of 2019, diabetes prevalence was estimated to be around 9.3%, affecting 463 million people. It is also expected to rise to 10.2% by 2030 which would correlate to 578 million people [2]. This widespread global burden makes diabetes a major focus of medical research. The treatment of diabetes was drastically changed by the discovery of insulin in 1922, and patients were now able to lower their blood glucose with insulin [3]. Since 1922, there have been numerous advancements for diabetic patients. For example, glucose monitoring technology allows patients to have greater glycemic control [4].

During this century, novel glucose-lowering agents have been developed for Type 2 diabetes such as sodium–glucose cotransporter-2 inhibitors such as dapagliflozin [5]. The clinical significance of SGLT2is has been established through multiple large-scale randomized controlled trials demonstrating both renal and cardiovascular benefit. The DAPA-HF trial showed that dapagliflozin significantly reduced the risk of worsening heart failure and cardiovascular death in patients with heart failure with reduced ejection fraction, regardless of diabetic status [6]. Similarly, the CREDENCE trial demonstrated that canagliflozin reduced the risk of end-stage kidney disease and renal outcomes in patients with diabetic nephropathy [7]. These landmark trials highlight the transition of SGLT2 inhibitors from glucose-lowering agents to disease-modifying therapies across multiple organ systems.

Mechanistically, the benefits of SGLT2 inhibition extend beyond glycosuria and involve coordinated effects on renal hemodynamics, tubular transport processes, inflammatory signaling, cellular metabolism, and oxidative stress. In particular, emerging evidence highlights the ability of SGLT2is to modulate epithelial transport proteins, regulate intracellular signaling pathways, and protect against structural and functional damage within the kidney. These pleiotropic effects provide a mechanistic foundation for their observed clinical efficacy.

This review focuses on the expanding understanding of SGLT2i biology, with particular emphasis on dapagliflozin as a model agent. We examine the historical development of SGLT inhibition, the recognized mechanisms of glucose transport, and the growing body of evidence supporting glucose-independent effects in renal, cardiovascular, and potentially pulmonary systems. By integrating findings from molecular, cellular, and clinical studies, this review aims to provide a comprehensive perspective on the evolving role of SGLT2is as systemic regulators of organ function.

### Historical Perspective of SGLTis

The first known SGLT inhibitor, phlorizin, was discovered by scientists in 1835, who isolated the product from the bark of an apple tree [8]. Subsequent experiments were designed to promote understanding of the physiology of phlorizin. These physiological investigations elucidated that phlorizin induced glycosuria was mediated by inhibition of glucose transport in the kidneys [9,10]. These findings initially positioned phlorizin as a promising candidate for hyperglycemia management. However, limitations soon emerged. Phlorizin’s broad mechanism of action and poor oral bioavailability posed significant challenges. When administered intravenously, it completely induced glycosuria, but data showed that large oral doses of phlorizin produced only short periods of partial glycuresis accompanied by gastrointestinal symptoms including nausea [11]. This warranted the belief that oral administration of phlorizin was substantially less effective than intravenous delivery. Mechanistic studies further revealed that phlorizin competitively inhibited intestinal glucose uptake at the brush-border membrane of the small intestine [12]. Beyond gastrointestinal effects, phlorizin was shown to mediate additional inhibitory pathways with detrimental physiological consequences. Administration of phlorizin was found to also inhibit glucose transport from the blood into the brain, although the mechanism was incompletely understood [13]. Together, these findings indicated that despite its glycosuria inducing capacity, phlorizin’s non-selectivity and systemic adverse effects precluded its clinical use as an antidiabetic therapy. This directed renal glucose transport inhibition research towards alternative approaches.

**Figure 1:**
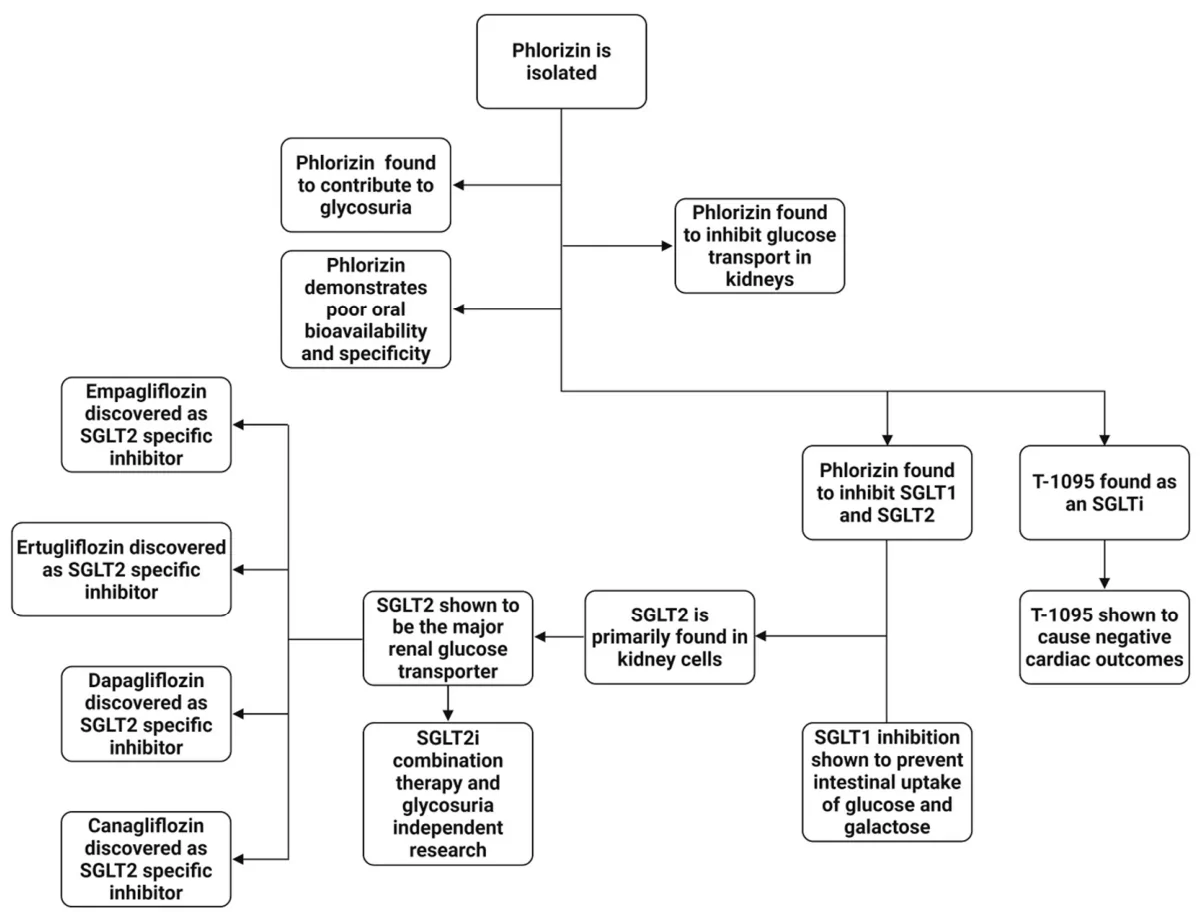
Timeline of key discoveries leading to the development of sodium glucose cotransporter inhibitors (SGLTis). The diagram outlines the progression from the isolation of phlorizin and its identification as a glucose transport inhibitor, through the characterization of SGLT subtypes, to the development of selective SGLT2 inhibitors currently used in clinical practice. Figure created with BioRender (https://www.biorender.com/).

The molecular basis of sodium–glucose co-transport was clarified decades later. Through expression cloning, SGLT1 was discovered in 1987 [14]. A few years later in 1994, homology screening revealed the presence of SGLT2 [15]. These breakthroughs provided the foundation for more targeted research into glucose reabsorption pathways in the kidney and small intestine. Sequence analysis of amplified SGLT1 complimentary DNA (cDNA) revealed the role of intestinal SGLT1 in 1991. SGLT1 cDNA provided evidence that SGLT1 is the primary carrier of glucose and galactose in the intestine [16]. Not long after, it became evident that phlorizin exhibited non-selective inhibition, targeting both SGLT1 and SGLT2 transporters [17,18]. The revelation of SGLT1 inhibition by phlorizin explains previous discoveries of gastrointestinal effects. SGLT1 presence in the intestine enabled phlorizin to inhibit intestinal glucose uptake, hence contributing to the symptoms observed. In 1995, in situ hybridization studies localized SGLT2 expression specifically to the S1 segments of the renal proximal tubule, highlighting its critical role in renal glucose reabsorption [19]. A phlorizin derivate, T-1095, was discovered as a potential antidiabetic agent in 1999. This novel SGLTi inhibited glucose uptake in various kidney types. Compared to phlorizin, T-1095 could be administered orally rather than intravenously [20]. However, subsequent studies revealed, like phlorizin, T-1095 demonstrated non-selective SGLT1 inhibition. This non-selective inhibition extended to SGLT1 expressed in cardiac tissue, potentially worsening cardiac outcomes following myocardial infarction [21]. Targeted research into SGLT2 function revealed that mutations in the SGLT2 gene were prevalent in patients exhibiting familial renal glycosuria, thereby confirming SGLT2’s dominant role in glucose reabsorption within the S1 and S2 segments of the proximal convoluted tubule [22]. The strong and selective nature of SGLT2 provided a clinically significant opportunity for antidiabetic treatment. This research led to the development and clinical approval of several highly selective SGLT2 inhibitors, including empagliflozin [23], dapagliflozin [24], canagliflozin [25], and ertugliflozin [26], each of which demonstrated efficacy in lowering blood glucose via enhanced glycosuria [23,24,25,26]. SGLT2 specific inhibitors have created a multitude of opportunities beyond glycemic control. For example, a clinical trial involving patients presenting with chronic kidney disease, both with and without T2DM, demonstrated dapagliflozin’s capability to lower the risk of death from renal or cardiovascular causes [27]. These findings have shifted research priorities toward understanding both glycosuria-dependent and glycosuria-independent mechanisms of benefit. Recently, SGLT2is are being explored in combination therapies with other antidiabetic agents, which will be discussed later in this paper. From the isolation of phlorizin in the 19th century to the advancement of modern SGLT2is, over 180 years of research have transformed a plant extract into a diverse class of drugs with proven benefits in T2DM, Chronic Kidney Disease (CKD), and potentially other metabolic and cardiovascular diseases or conditions such as heart failure (HF). This progression is summarized in Figure 1.

## 2. Mechanism of Action

**Figure 2:**
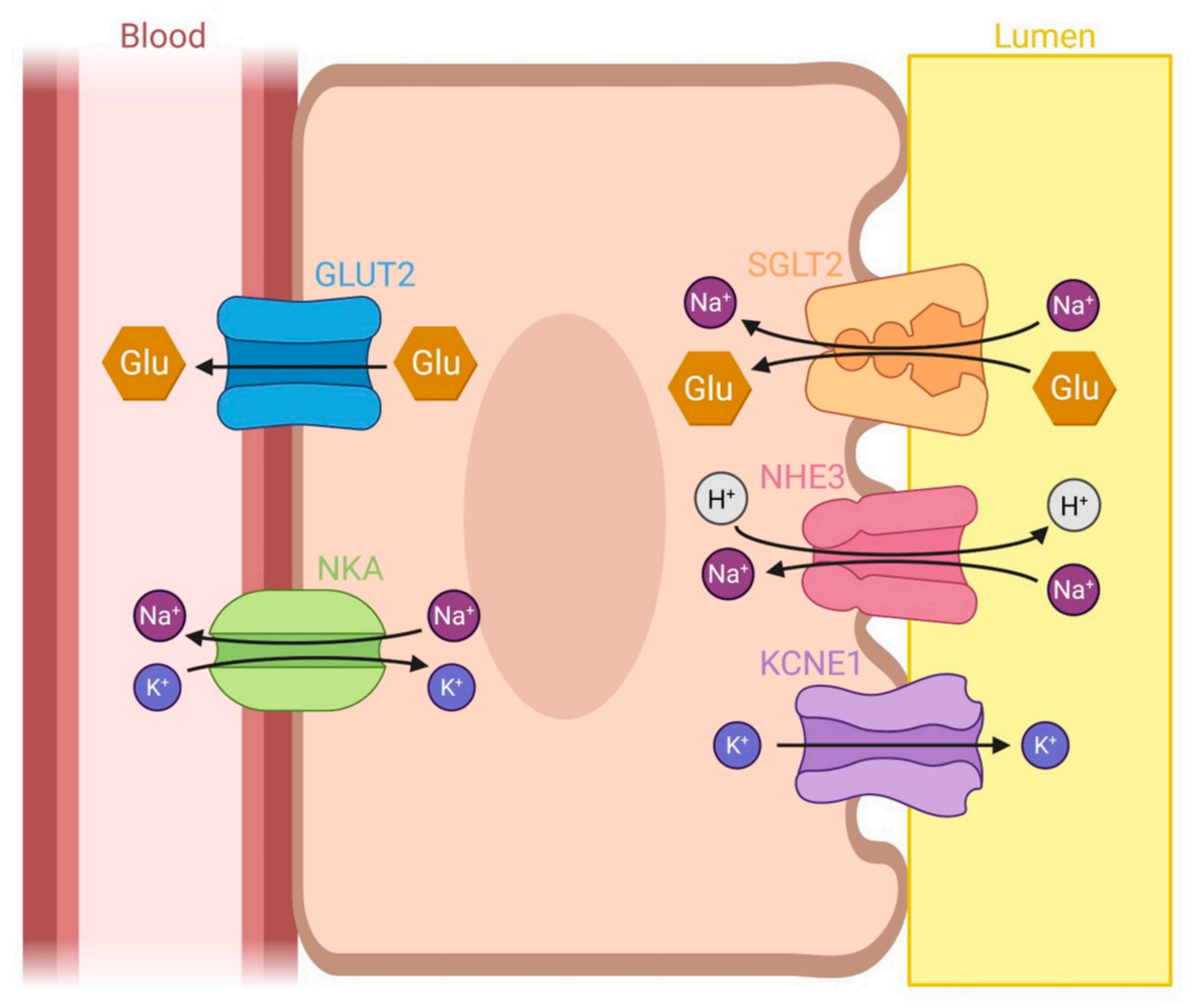
Coordinated glucose and ion transport across the renal proximal tubule epithelium. SGLT2 on the apical brush-border membrane cotransports Na^+^ and glucose from the tubular lumen into proximal tubule cells, generating an electrogenic depolarization that is stabilized by KCNE1-mediated K^+^ efflux. Intracellular glucose then exits across the basolateral membrane via GLUT2, while Na^+^ is recycled by the Na^+^/K^+^-ATPase (NKA), which maintains the electrochemical gradient necessary for continued Na^+^-dependent glucose uptake. To sustain high rates of transport, proximal tubule cells rely on ATP generated through oxidative metabolism, producing protons that must be exported to preserve intracellular pH. This proton extrusion is carried out by the apical Na^+^/H^+^ exchanger NHE3. Together, these transporters function in an integrated manner to reabsorb filtered glucose and maintain ionic and pH homeostasis. Figure created with BioRender (https://www.biorender.com/).

SGLT2 is a protein that serves as a sodium–glucose cotransporter. This protein is localized to the brush border membrane (BBM) of the renal proximal tubule, predominantly expressed in the S1 segment and to a lesser extent in the S2 segment, while being absent in segment S3 [28]. A total of 97% of filtered glucose is reabsorbed by SGLT2 while the remaining 3% of filtered glucose is reabsorbed in segments S2 and S3 by SGLT1 [29]. This is what makes SGLT2 the major glucose transporter and hence the target of diabetic treatment. Functionally, SGLT2 operates as a high-capacity, low affinity Na^+^-D-glucose cotransport system, exhibiting a 1:1 stoichiometry in which one sodium is co-transported with each glucose molecule [30]. This process is electrogenic, as the inward movement of positively charged sodium ions with electrically neutral glucose depolarizes the luminal cell membrane. The resulting depolarization is attenuated by KCNE1 through potassium efflux across the membrane, maintaining electrochemical stability [31]. Na^+^/K^+^ ATPase (NKA) is another protein that stabilizes the electrochemical gradient needed for glucose transport. NKA is located on the basolateral membrane and uses counter-transport of Na^+^ and K^+^ to allow Na^+^ to enter the cell through SGLT2 [32]. Along with sodium, SGLT2 transports glucose across the BBM and then works with glucose transporter GLUT2, located on the basolateral membrane of cells [33]. After the glucose and sodium are moved into the cell, GLUT2 transports glucose from the tubular epithelium into the peritubular vasculature [34]. GLUT2 is a major renal transporter of glucose into the blood stream which contributes to hyperglycemia observed in diabetes [35]. Together, these coordinated transport processes ensure efficient transcellular movement of glucose from the tubular lumen into the bloodstream and maintain the electrochemical balance required for this task. Because each transporter relies heavily on ATP generated through oxidative metabolism [36], proton by-products must be continuously removed to preserve intracellular pH [36,37]. This is accomplished by the apical Na^+^/H^+^ exchanger NHE3, which exports protons in exchange for sodium, thereby supporting ongoing Na^+^-dependent glucose reabsorption [38]. NHE3 should be emphasized as a central proximal tubule sodium transporter rather than a secondary supporting exchanger. In experimental NHE3-deficient mice, disruption of NHE3 activity resulted in significantly decreased Na^+^ and HCO$3^{-}$ absorption, supporting its major role in epithelial sodium and acid-base transport [37]. In the proximal tubule, this exchanger works by coupling its own Na^+^ uptake to the extrusion of Na^+^ by basolateral NKA [39]. Therefore, increased proximal tubule sodium–glucose transport in diabetes is not an isolated SGLT2 event but occurs within a broader transport network involving NHE3, NKA, and bicarbonate reclamation. As illustrated in Figure 2, the integrated actions of SGLT2, GLUT2, NKA, NHE3, and associated ion channels sustain renal glucose handling and highlight why modulation of SGLT2 has emerged as an effective therapeutic strategy for type 2 diabetes.

### 2.1. SGLT2 Structure

To further understand SGLT2 mechanics, it is important to recognize the structural elements involved in these processes. The sodium–glucose transporter family is made up of sodium-coupled transporters that are structurally similar to SGLT1. Although their transported solutes vary, all members of the SGLT family share a common core of 13 transmembrane helices [40]. The SLC5 family of genes are responsible for coding the intestinal and renal glucose transporters [41]. Specifically, the gene SLC5A2 is responsible for translating the SGLT2 protein [42]. Along with the SGLT common core of 13 transmembrane helices (TM), SGLT2 has an additional helix totaling 14 transmembrane helices. Among these, TM1-TM5 and TM6-TM10 are structurally similar but show topologically inverted repeats (IRs). The transporter core is made up of these two IRs [43]. In order to transport glucose, SGLT2 requires the accessory protein MAP17 [44]. During glucose transport, SGLT2 changes between outward and inward facing conformations where sodium binds to the outward conformation. MAP17 helps to stabilize SGLT2 during these alternating conformations, allowing glucose reabsorption to take place [45]. These elements contribute to SGLT2’s innate ability to reabsorb glucose; however, they also create an opportunity for inhibition and altered glycemic control.

### 2.2. Dapagliflozin Mechanism of SGLT2 Inhibition

SGLT2is are glucosides composed of a sugar moiety linked to an aromatic tail known as an aglycon. As discussed previously, SGLT2 undergoes conformational cycling between outward- and inward-facing states. Phlorizin binds to both SGLT1 and SGLT2 in the outward facing conformation through aromatic interactions mediated by its aglycon tail. A notable difference between SGLT1 and SGLT2 is that SGLT2 has a third aromatic residue, H268, that creates an aromatic cage. Rather than an aromatic amino acid, SGLT1 has an aspartic acid at position 268 and no aromatic cage is formed. Instead, D268 of SGLT1 creates a salt bridge with R267 facilitating a cation-π interaction with bound inhibitors. While phlorizin’s central aromatic ring can interact with the salt-bridge environment of SGLT1, dapagliflozin binds much weaker because its central ring sits deeper within the binding pocket and cannot engage R267 effectively. In contrast, the aromatic cage formed by H268 in SGLT2 accommodates dapagliflozin and stabilizes binding, explaining the drug’s roughly 100-fold higher selectivity for SGLT2 over SGLT1 [46]. Dapagliflozin administration is associated with reduced blood pressure [47], body weight, glomerular filtration rate, and elevated glycemic control making this drug the focus of our review [48]. Other SGLT2is such as empagliflozin, canagliflozin, and ertugliflozin have demonstrated beneficial effects like dapagliflozin; but studies have shown that, despite their structural similarity, differences still exist.

### 2.3. Comparative Pharmacokinetics of SGLT2 Inhibitors

Since SGLT inhibitors are structurally similar, comparisons can be made regarding their pharmacokinetics instead. For example, empagliflozin exhibits higher selectivity for SGLT2 over SGLT1 compared to the rest of the inhibitors [23]. This selectivity is particularly important because of the previously discussed gastrointestinal issues associated with SGLT1 inhibition. Another study found that although all SGLT2 inhibitors produced beneficial effects through weight loss reduction, canagliflozin was the most successful at lowering weight in T2DM patients [49]. The primary purpose of SGLT2 inhibitors is to induce glycosuria so comparisons between those levels are critical. When analyzing urine glucose excretion by healthy volunteers, empagliflozin administration produced 30.6–78.6 g/day, dapagliflozin produced 60 g/day, and canagliflozin produced 59 g/day [50]. Beyond diabetic kidney disease, SGLT2 inhibitors have demonstrated substantial benefits in chronic kidney disease independent of glycemic status. The DAPA-CKD trial showed that dapagliflozin significantly reduced the risk of sustained decline in kidney function, end-stage kidney disease, and renal or cardiovascular death in patients with CKD both with and without diabetes [27]. Similarly, the EMPA-KIDNEY trial demonstrated that empagliflozin reduced progression of kidney disease and cardiovascular death across a broad CKD population, including non-diabetic patients [51]. These findings confirm that the renoprotective effects of SGLT2 inhibitors extend beyond glucose lowering, which will be discussed later in this review. Although empagliflozin exhibits higher selectivity, it has also been found to raise LDL cholesterol levels [52]. Another study found that ertugliflozin and canagliflozin produced similar increases in LDL levels [26]. Increased LDL levels can raise atherogenic risk; however, treatment with dapagliflozin did not show the increased LDL levels observed with other SGLT2is [53]. When looking at patients with T2DM, the target group of dapagliflozin, having an increase in LDL cholesterol has been shown to be a risk factor for coronary artery disease [54]. Conversely, a reduction in LDL is directly proportional to a decrease in major cardiovascular events [55]. Importantly, dapagliflozin’s cardiovascular relevance should not be interpreted solely through changes in LDL levels. Rather, its lipid-related benefits appear to involve broader remodeling of the cardiometabolic environment. In overweight patients with T2DM insufficiently regulated by metformin, dapagliflozin significantly reduced body weight, total fat mass, and both visceral and subcutaneous adipose tissue, suggesting that its effects extend beyond glycosuria to clinically meaningful changes in adiposity [56]. Overall, reduction in fat is obviously beneficial for a T2DM patient’s cardiovascular health, but dapagliflozin may improve lipid-associated cardiovascular risk indirectly through reduced adiposity and improved cardiometabolic substrate handling, even when LDL cholesterol changes are neutral or modest. This suggests a unique therapeutic advantage for dapagliflozin to impact cardiovascular health and potentially other organ systems, which is why this SGLT2 inhibitor is the focus of our review. Although pharmacokinetic differences distinguish individual SGLT2is, these properties alone do not fully explain their broad clinical benefits. Increasing evidence points toward additional mechanisms that extend beyond glycemic control. These observations suggest that dapagliflozin influences cellular metabolism at multiple levels, including lipid handling, inflammatory signaling, and energy utilization, mechanisms that likely contribute to its systemic protective effects.

### 2.4. Tubuloglomerular Feedback

A crucial impact of SGLT2 inhibition beyond glucose transport is the significant alteration in renal hemodynamics through the restoration of tubuloglomerular feedback (TGF). For patients with DKD, diabetes is a risk factor for end-stage renal disease [57]. Patients with end-stage renal disease experience fluid overload, which significantly increases the risk of mortality [58]. Under diabetic conditions, excessive proximal tubular sodium and glucose reabsorption reduces sodium chloride delivery to the macula densa, impairing TGF signaling and promoting glomerular hyperfiltration [59]. Treatment with SGLT2 inhibitors, particularly empagliflozin, reduces hyperfiltration by decreasing sodium/glucose reabsorption along the proximal tubule, resulting in increased sodium chloride transport to the distal tubule [60]. Similarly, dapagliflozin-induced increases in glucose and sodium excretion produce a diuretic effect, associated with elevated plasma renin activity, increased serum aldosterone, and ultimately a decrease in plasma volume [48]. SGLT2is can also impact NHE3 activity and mitochondrial function, resulting in altered tubuloglomerular feedback. Canagliflozin was shown to inhibit NHE3 and reduce mitochondrial respiration, thereby inhibiting albumin uptake and fluid transport [61]. In the proximal tubule, NHE3 is involved in salt and water reabsorption [62]. As discussed earlier, NHE3 is coupled to the ATPase NKA. ATPases release adenosine, which restricts afferent arterioles, decreasing glomerular pressure and GFR [63]. Hence, the decrease in NHE3 activity from SGLT2 inhibition contributes to the positive effect on renal hemodynamics. Overall, the restoration of TGF is believed to play a central role in the renoprotective effects of SGLT2 inhibitors by reducing mechanical stress on the glomerulus and slowing progression of CKD. Importantly, these hemodynamic effects occur independently of glucose lowering and help explain the clinical benefits observed in both diabetic and non-diabetic populations [27].

### 2.5. SGLT Inhibition and Its Role in Renal Physiology

The focus of this paper is dapagliflozin and its effects beyond the reduction in proximal tubule glucose reabsorption, so it is important to first understand the impact of SGLT2is on the renal system. As discussed previously, the prevalence of diabetes contributes to the progression of CKD, along with other complications. However, studies have found that SGLT2is may provide renal protection for patients with CKD, even in cases of stage 5 CKD and without causing adverse cardiovascular effects [64]. Additional data suggest that these renoprotective effects, through attenuation of diabetic nephropathy progression, are attributed to blood glucose-dependent and -independent mechanisms. These mechanisms include diminishing diabetic induction of albuminuria, kidney growth, and renal expression of molecular markers of kidney inflammation [65]. The decrease in albuminuria is particularly significant since albuminuria is a biomarker of nephropathy progression. Specifically, studies have found that in T2DM patients with increased albuminuria, SGLT2i administration reduces the progression of end-stage kidney disease [66]. These results suggest that SGLT2is offer renoprotective effects by lowering renal glucose reabsorption, along with reductions in nephropathy through altered protein handling. The role of SGLT2 inhibition in renal physiology goes well beyond the mechanisms discussed in this subsection, but it is necessary to appreciate the renal benefits mentioned to fully grasp the systemic impacts discussed in this paper.

## 3. Mechanisms of Dapagliflozin Beyond Glucose Transport

Given the established mechanisms of glucose handling, attention has shifted toward understanding how SGLT2is exert protective effects independent of glycosuria. One study showed dapagliflozin inhibits macrophage-mediated inflammation independent of SGLT2 and that this mechanism protects against chronic heart failure [67]. Another study reported that administration of dapagliflozin to hypertensive diabetic db/db mice causes an increase in the amount of bioactive phosphatidylethanolamine in kidney membrane fractions and alters the density of lipid rafts in mouse proximal tubule cells [68]. These findings illustrate the diverse functional effects of dapagliflozin across multiple biological systems.

### 3.1. Dapagliflozin Treatment Alters Expression of Epithelial Transport Proteins in the Kidney

**Figure 3:**
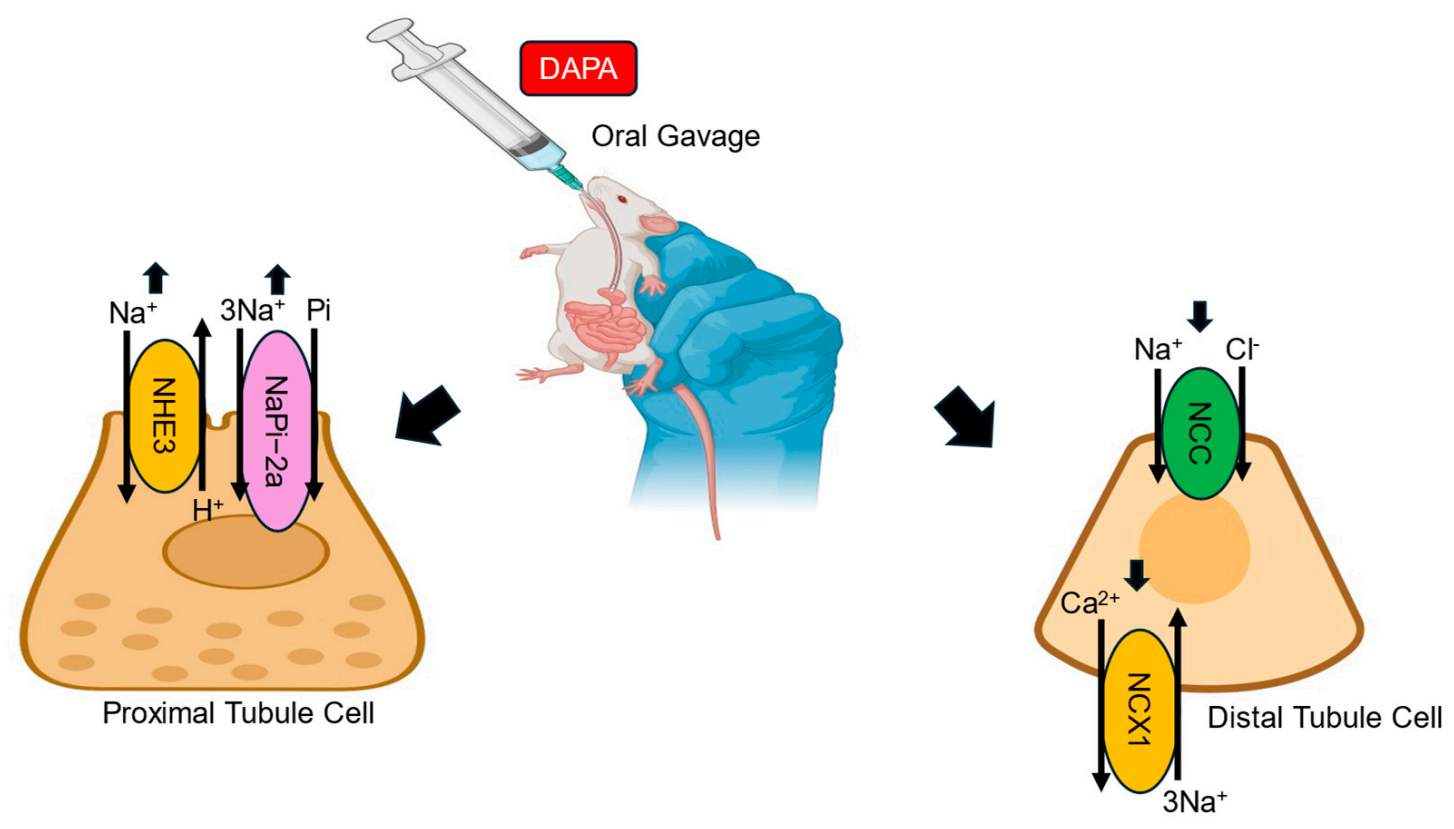
Western blot analysis from a study by Ma et al. showed that NaPi-2a and NHE3 protein expression are upregulated in the kidneys of diabetic mice given an oral gavage of dapagliflozin when compared to vehicle saline treated mice [69]. Also, mRNA results from this study showed NCX1 is decreased in the kidney. Western blot and immunohistochemistry analysis from a study by Gholam and Alli showed protein expression phospho-NCC and total NCC is decreased in the kidney db/db mice administered dapagliflozin by oral gavage compared to vehicle saline [47]. Sodium chloride co-transporter (NCC); Na^+^-hydrogen antiporter 3 (NHE3); Na^+^/phosphate cotransporter (NaPi-2a). Figure created with BioRender (https://www.biorender.com/).

Multiple studies have investigated the effects of dapagliflozin on the expression of transmembrane proteins involved in transport in the kidneys of diabetic mice. One study by Ma et al. [69] used a streptozotocin induced diabetic mouse model to investigate the effects of oral gavage administration of dapagliflozin compared to vehicle for 18 days on epithelial transport proteins (Figure 3). Their protein analysis showed NaPi-2a and NHE3 are upregulated in dapagliflozin-treated diabetic mice while their mRNA analysis showed NCX1 expression declined in the dapagliflozin group. A different study investigated the effects of dapagliflozin administration by oral gavage for 14 days in salt-loaded hypertensive diabetic db/db mice. Immunohistochemistry results were consistent with Western blot and densitometric analysis of total NCC and phospho-NCC being downregulated in the dapagliflozin treated group [47]. Mechanistically, other data from this study revealed that dapagliflozin treatment reduces sodium retention, normalizes blood pressure, and reduces abnormally high levels of total and phospho-NCC by reducing the interaction between NCC and the actin cytoskeleton linker protein ezrin [47]. Although these studies focus on dapagliflozin, it is suggested that these mechanisms are conserved across the SGLT2 inhibitor class. Because abnormal sodium handling and glomerular hyperfiltration are major drivers of CKD progression, modulation of these transport proteins may contribute directly to the long term renoprotective effects observed clinically with SGLT2 inhibition.

### 3.2. Dapagliflozin Inhibits Podocyte Injury and Cell Death

Podocyte injury is a central driver of albuminuria and the progression of diabetic kidney disease (DKD) [70], making it a critical therapeutic target. Emerging evidence demonstrates that dapagliflozin exerts direct protective effects on podocytes through coordinated regulation of inflammatory, metabolic, and cell survival pathways. A key mechanism underlying this protection involves suppression of inflammatory and pyroptotic signaling. In a recent study, Zhang et al. used streptozotocin-induced type 2 diabetic mice to investigate how dapagliflozin mitigates diabetes-related kidney damage [71]. Mice in the treatment group received dapagliflozin for six weeks, after which renal tissue was examined for markers of pyroptosis. Western blot analysis revealed that the diabetic control group exhibited elevated levels of NLRP3, GSDMD-N, caspase-1, ASC, and the inflammatory cytokines IL-18 and IL-1β. These increases were markedly reduced in the dapagliflozin-treated mice. Immunofluorescence and qPCR results further confirmed that dapagliflozin suppressed the diabetes-induced upregulation of these pyroptotic and inflammatory factors. Further mechanistic investigation showed that miR-155-5p expression was downregulated in diabetic mice. Inhibition of miR-155-5p induced pyroptosis, decreased HO-1 expression, and increased NLRP3 expression, highlighting its involvement in the protective pathway activated by dapagliflozin [71]. In addition to inflammatory signaling, dapagliflozin also influences intracellular signaling pathways associated with cellular stress and structural remodeling. A study by Guo et al. also focused on the mechanism by which dapagliflozin produces its renoprotective effects by using streptozotocin-induced diabetic mice with dapagliflozin treatment compared to diabetic and non-diabetic controls [72]. Transcriptomic analysis of renal tissue showed that IGF1R and PI3K signaling pathways were markedly upregulated in the diabetic group, whereas dapagliflozin treatment significantly suppressed their expression. Complementary cell-culture experiments further confirmed this relationship: high-glucose exposure increased IGF1R and PI3K signaling, while the selective IGF1R inhibitor OSI-906 reduced PI3K activation, demonstrating their functional connection. Together, these findings reveal that SGLT2, IGF1R, and PI3K work together to regulate epithelial–mesenchymal transition and contribute to dapagliflozin’s protective effects against diabetic renal injury [72]. Given that podocyte dysfunction and epithelial–mesenchymal transition contributes to progressive albuminuria and glomerulosclerosis, suppression of these pathways may represent an important mechanism through which dapagliflozin preserves renal filtration integrity. Autophagy also plays a critical role in maintaining podocyte homeostasis, and its impairment contributes to diabetic kidney injury. Feng et al. conducted a study where mice were divided into three groups: a normal diet control, a high-fat diet (HFD) to induce diabetes, and a high-fat diet combined with dapagliflozin treatment to assess its renoprotective effects in relation to autophagic activity [73]. Immunohistochemical analysis of renal tissue revealed significantly increased expression of HMGB1, a pro-inflammatory mediator, in the diabetic group compared to controls. This upregulation of HMGB1 was notably reduced by dapagliflozin treatment. Additionally, the diabetic kidneys exhibited suppressed levels of the autophagy marker LC3A/B and increased accumulation of P62, indicating impaired autophagic flux associated with renal injury. Treatment with dapagliflozin reversed these changes, restoring autophagy and decreasing P62 accumulation. These findings highlight dapagliflozin’s dual role in inhibiting HMGB1-driven inflammation and rescuing autophagy dysfunction, thereby contributing to its protective effects against diabetic kidney damage [73]. At the functional level, these molecular changes translate into measurable reductions in renal injury biomarkers. A study by Oraby et al. investigated whether dapagliflozin could alleviate streptozotocin-induced diabetic renal injury in rats and compared its effects with metformin, a traditional antidiabetic, alone and in combination therapy [74]. Diabetic rats showed a 10.5-fold increase in urinary N-acetyl-β-D-glucosaminidase (NAG), indicating significant tubular injury. Dapagliflozin reduced NAG levels, while the combination of dapagliflozin and metformin restored NAG to normal control values. Enzyme linked immunosorbent assay (ELISA) analysis revealed that renal injury markers kidney injury molecule 1 (KIM-1), neutrophil gelatinase-associated lipocalin (NGAL), and cystatin C were all elevated in diabetic rats. Dapagliflozin significantly lowered KIM-1 and NGAL, although cystatin C decreased only with the combination therapy. Additionally, diabetic rats exhibited elevated malondialdehyde (MDA), a marker of oxidative stress, which was reduced by either dapagliflozin alone or combination therapy. Collectively, these findings indicate that dapagliflozin attenuates diabetic renal injury by reducing KIM-1, NGAL, NAG, and MDA levels, with even greater protective effects observed when combined with metformin [74]. Additional evidence highlights the role of stress-response proteins such as REDD1 in mediating inflammatory and pyroptotic injury. An animal study by Sunilkumar et al. investigated the role of REDD1 in diabetic pathology [75]. REDD1 wild-type (REDD1^+^/^+^) and REDD1 knockout (REDD1^−^/^−^) mice were treated with streptozotocin to induce diabetes while the control group received vehicle. Following treatment with dapagliflozin, diabetic mice exhibited markedly reduced REDD1 protein abundance and decreased immune-cell infiltration compared with untreated diabetic controls. These findings were supported by mRNA expression analysis, which showed that REDD1^+^/^+^ mice displayed elevated expression of inflammatory mediators compared to REDD1^−^/^−^ mice. Thus, the normalization of blood glucose achieved with dapagliflozin appears to lower REDD1 protein levels, contributing to diminished inflammatory cell recruitment. Western blotting determined the role of REDD1 expression in podocytes to contribute to NF-KB and NLRP3 activation and associated pyroptotic cell death [75]. Collectively, these findings demonstrate that dapagliflozin protects podocytes through multi-level regulation of inflammatory signaling, autophagy, oxidative stress, and cellular survival pathways. Rather than acting solely through glycosuria, dapagliflozin engages a network of intracellular mechanisms that preserve podocyte structure and function, ultimately contributing to reduced albuminuria and slowed progression of DKD. The benefits of this medication, and similar medications of its class, extend far beyond the original purpose of SGLT2 inhibition. SGLT2is provide promising possibilities in the clinical setting suggesting how further research outside proximal tubule cells role can contribute to the understanding of the full capacity of this drug class.

### 3.3. Dapagliflozin’s Effects on Signaling Pathways in Proximal Tubule Cells

Emerging evidence indicates that SGLT2 inhibitors protect proximal tubular epithelial cells through coordinated suppression of inflammatory, fibrotic, and stress-response signaling pathways. Studies using human proximal tubule (HK-2) cells have shown that dapagliflozin inhibits various pathways in these cells. For example, dapagliflozin was shown to suppresses the receptor-interacting protein kinase 2 (RIPK2)–receptor-interacting protein kinase 3 (RIPK3)–mixed lineage kinase domain-like (MLKL) RIP2-RIP3-MLKL signaling axis in HK-2 cells treated with H_2_O_2_ [76]. Data from clinical studies have demonstrated that RIPK family members are significantly elevated in the plasma of patients with myocardial infarction while correlating with disease severity [77]. Moreover, these pathways are closely linked to tubular injury and inflammatory fibrosis, hence their suppression may help limit long term nephron loss during CKD progression. Another study showed dapagliflozin inhibits high glucose-induced activation of YAP/TAZ in HK-2 cells [78]. Because YAP/TAZ signaling contributes to epithelial–mesenchymal transition and extracellular matrix accumulation, suppression of this pathway may help limit progressive renal fibrosis. The systemic targeting of YAP/TAZ in patients is novel, and emerging data suggests there are distinct and manageable side effects. Experiment evidence demonstrated that dapagliflozin reduces the expression of renal fibrosis-related markers in HK-2 cells stimulated by TGF-β1 [79]. Another study reported that in HK-2 cells, dapagliflozin and fludarabine directly decreased aberrant STAT1 expression [80]. Collectively, these findings suggest dapagliflozin protects tubular epithelial cells by suppressing maladaptive stress signaling pathways associated with CKD progression.

### 3.4. Dapagliflozin Ameliorates Renal Fibrosis Through the Inhibition of Signal Transduction Pathways

**Figure 4:**
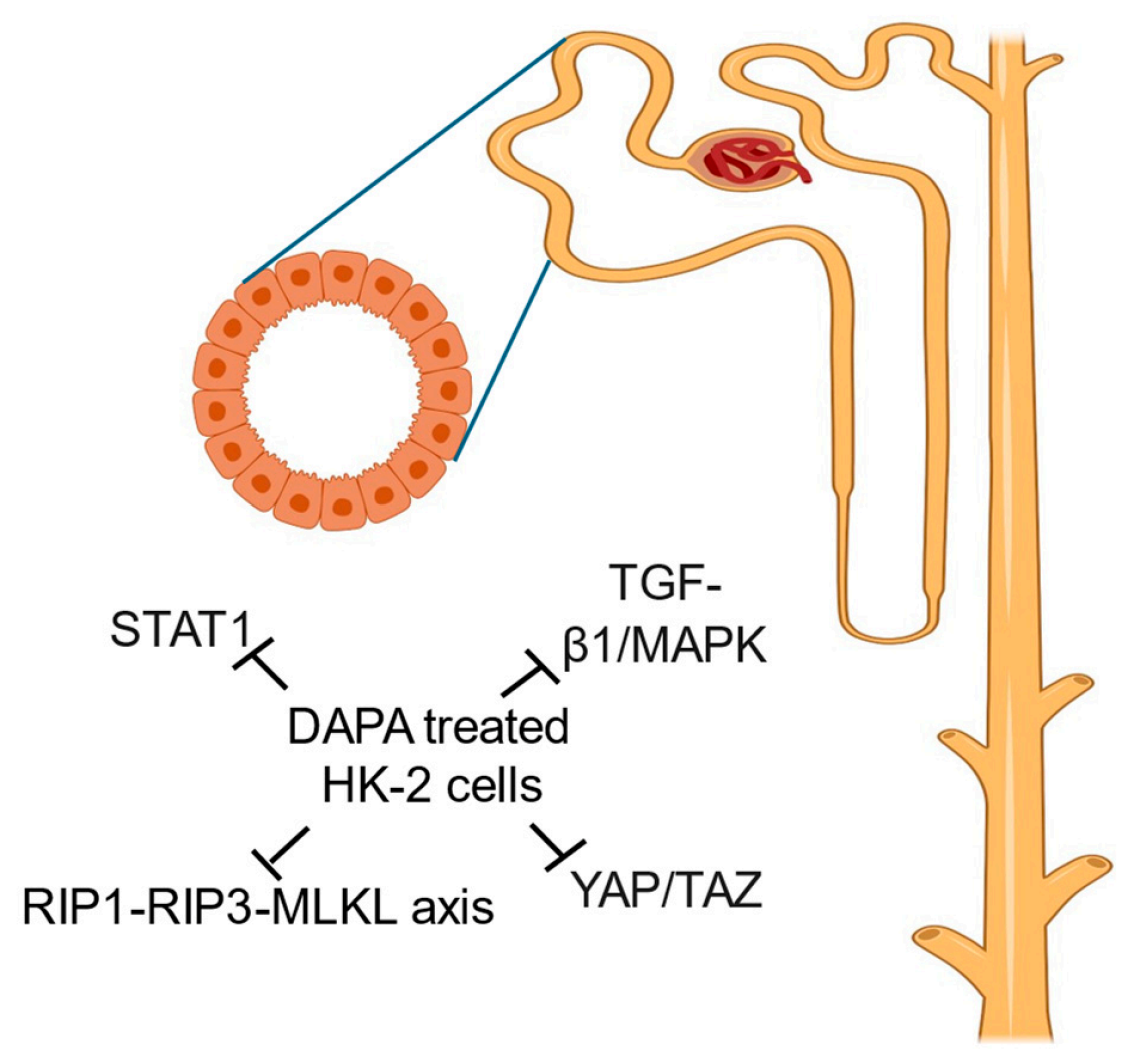
Effect of dapagliflozin in proximal tubule cells. Dapagliflozin treatment alters various signaling pathways in human proximal tubule (HK-2) cells. Dapagliflozin treatment has been shown to either decrease or inhibit STAT1 [80], YAP/TAZ [78], RIP1-RIP3, MLKL [76], and TGF-β1 [79]. Figure created with BioRender (https://www.biorender.com/).

**Figure 5:**
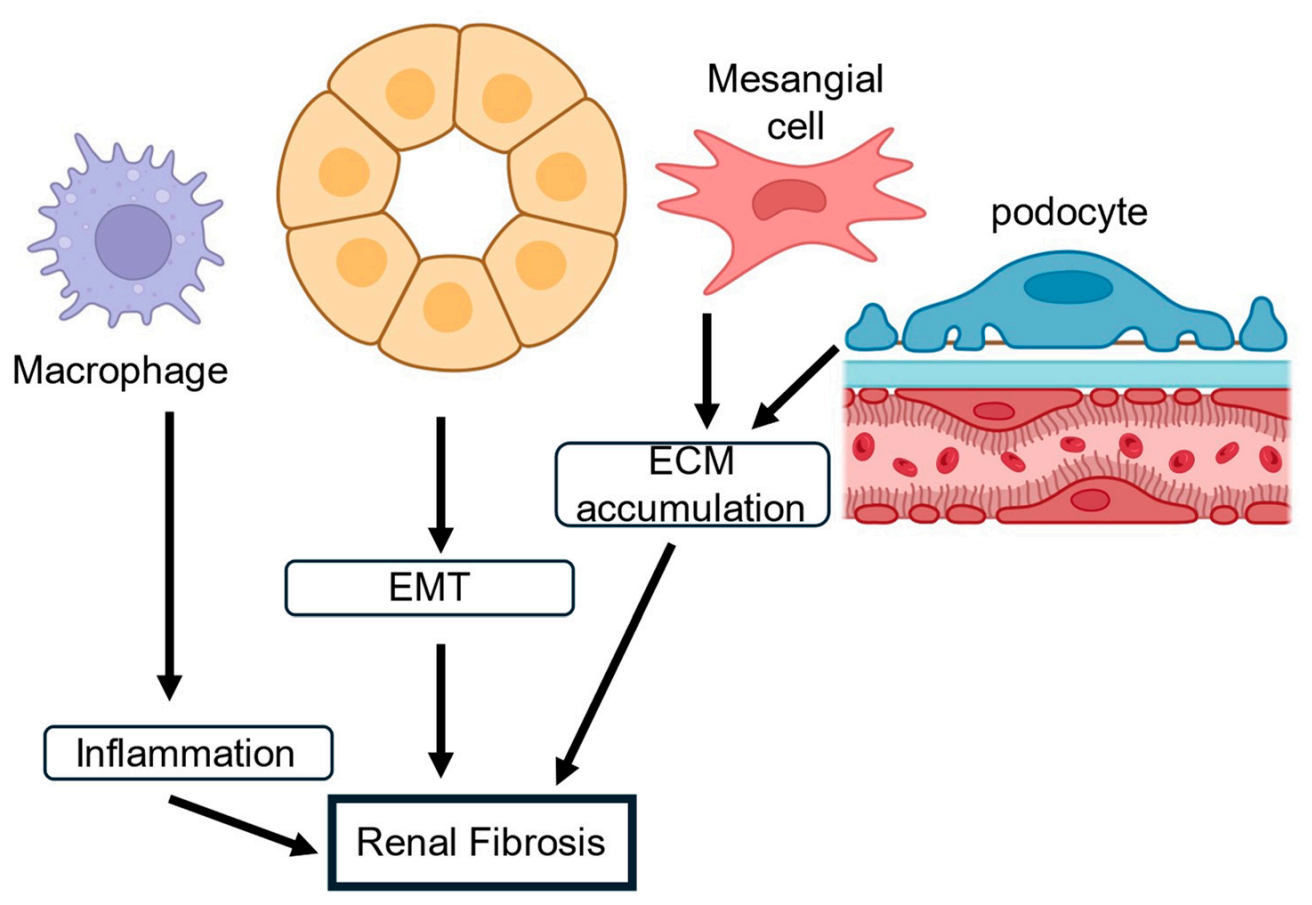
Dapagliflozin attenuates renal fibrosis at multiple levels. Dapagliflozin alters signal transduction pathways involved in inflammation, epithelial–mesenchymal transition (EMT), and accumulation of the extracellular matrix to suppress the progression of renal fibrosis. Figure created with BioRender (https://www.biorender.com/).

Renal fibrosis characterized by the accumulation of extracellular matrix, activation of fibroblasts, tubule epithelial cell apoptosis, and infiltration of inflammatory cells are some of the common signs of progressive CKD. As shown in Figure 4, several groups have shown dapagliflozin attenuates renal fibrosis by inhibiting various signaling transduction pathways. Dapagliflozin has been shown to attenuate renal fibrosis in a mechanism involving the inhibition of the RIP1-RIP3-MLKL-mediated necroinflammation pathway in a model of unilateral ureteral obstruction (UUO) [76]. In another study dapagliflozin was shown to delay renal fibrosis in DKD in a mechanism involving the inhibition of the yes-associated protein (YAP)/transcriptional coactivator PDZ-binding motif (TAZ) in proximal tubule epithelial cells [78]. Another study showed dapagliflozin reduces renal fibrosis by suppressing the angiotensin II/TGFβ signaling pathway in diabetic mice [81]. A different study showed that dapagliflozin ameliorates renal fibrosis in a mouse model of adenine-induced renal injury through the inhibition of TGF-β1/MAPK mediated mitochondrial damage [79]. Another study reported dapagliflozin attenuates renal tubulointerstitial fibrosis type 1 diabetes by regulating the STAT1/TGFβ1 signaling pathway [80]. This is clinically significant because renal fibrosis represents a final common pathway in progressive CKD regardless of the initiating insult. As illustrated in Figure 5, dapagliflozin ameliorates the progression of renal fibrosis at least indirectly through the modulation of signal transduction pathways associated with inflammation, epithelial–mesenchymal transition (EMT), and accumulation of the extracellular matrix. Collectively, these mechanisms show the ability of dapagliflozin to decrease progress of CKD, which could benefit patients with and without diabetes. While dapagliflozin serves as the primary focus of this review, many of these mechanisms appear conserved across the SGLT2 inhibitor class.

Mitochondrial dysfunction is increasingly recognized as a central contributor to diabetic kidney disease, fibrosis, and cardiovascular injury. Emerging evidence suggests that dapagliflozin improves mitochondrial homeostasis through multiple complementary mechanisms, including preservation of mitochondrial membrane integrity and enhancement of cellular energetic efficiency in renal tissue [82]. In cardiac tissues, SGLT2 inhibition by empagliflozin has been associated with improved ATP production and altered metabolic substrate utilization, particularly increased ketone body oxidation, which may provide a more energy efficient fuel source under conditions of metabolic stress [83]. Additionally, treatment with dapagliflozin may contribute to reduced reactive oxygen species (ROS) production, enhanced cell viability, and modulation of autophagy-related proteins, linking mitochondrial preservation to the broader anti-inflammatory and antifibrotic effects of SGLT2 inhibitors [84].

### 3.5. Dapagliflozin Provides Cardioprotection Through Hemodynamic, Metabolic, and Molecular Mechanisms

Beyond renal effects, SGLT2 inhibitors exert significant cardiovascular benefits through integrated hemodynamic, metabolic, and molecular mechanisms. Large-scale clinical trials first revealed these benefits, and subsequent mechanistic studies have begun to clarify the molecular pathways involved. These cardioprotective actions are now understood to arise through a combination of improved cardiac energetics, suppression of inflammatory pathways, enhanced mitochondrial function, and favorable hemodynamic changes. One of the landmark trials establishing dapagliflozin’s cardiac benefits is the DAPA-HF trial, which showed that dapagliflozin significantly reduced the risk of cardiovascular death or worsening HF in patients with heart failure accompanied by reduced ejection fraction, independent of diabetic status [6]. Mechanistic studies have provided insight into the pathways through which these changes occur. Investigations show that dapagliflozin attenuates NLRP3 inflammasome activation in cardiofibroblasts, reducing downstream caspase-1 activation and levels of pro-inflammatory cytokines IL-1β and IL-6 [85]. By inhibiting NLRP3-mediated pyroptosis and oxidative stress, dapagliflozin protects cardiomyocytes from inflammation-driven injury. Other studies reveal improvements in mitochondrial efficiency, including enhanced ketone utilization, which improves cardiac work efficiency under stress [86]. Improved myocardial energetic efficiency may partially explain the rapid reductions in heart failure hospitalization observed in clinical trials. Additionally, dapagliflozin has been shown to reduce infarct size and decrease expression of myocardial inflammation-related proteins, protecting against myocardial ischemia/reperfusion injury through mechanisms involving the degradation of the NLRP3 inflammasome during autophagy [87]. Dapagliflozin has also demonstrated structural benefits in models of cardiac hypertrophy. In diabetic rodent models, dapagliflozin reduced myocardial hypertrophy, attenuated cardiac chamber dilation, and improved left ventricular dysfunction, suggesting a direct role in mitigating pathological cardiac remodeling [88]. Clinically, these actions are complemented by improvements in vascular endothelial function; dapagliflozin lowers oxidative stress in aortic rings and reduces arterial stiffness, which supports improved cardiac output [89]. Collectively, these studies highlight that dapagliflozin’s cardioprotective effects involve multifaceted pathways that extend well beyond glycemic lowering. Through coordinated improvements in hemodynamics, energy metabolism, oxidative stress, mitochondrial function, and inflammatory signaling, dapagliflozin consistently improves cardiac outcomes across diverse clinical and experimental settings. Importantly, these cardioprotective effects are not limited to dapagliflozin but represent a class-wide phenomenon supported by multiple landmark trials. The EMPA-REG OUTCOME trial demonstrated that empagliflozin significantly reduced cardiovascular mortality and hospitalization for HF in patients with type 2 diabetes [90]. The CANVAS Program showed that canagliflozin reduced major adverse cardiovascular events in a high-risk diabetic population, including HF [91]. Additionally, the VERTIS CV trial confirmed that ertugliflozin reduces hospitalization for HF while maintaining cardiovascular safety [92]. It is important to note how every SGLT2i mentioned was associated with reduced risk of HF. This class-wide effect explains why SGLT2is are now prescribed for HF, regardless of diabetic status [93]. Collectively, these studies support a consistent cardioprotective effect across SGLT2 inhibitors, with an extremely significant impact on HF reduction specifically. The decrease in HF observed is heavily owed to SGLT2i-induced benefits on intraglomerular hemodynamics. Dapagliflozin improves cardiac health by reprogramming mitochondrial function through restraint of renal oxygen consumption, rather than osmotic diuretic decongestion. This mechanism is caused by glucose loss in the urine leading to adaptive energy conservation while also requiring water retention to offset the osmotic diuretic effects of glycosuria [94]. Importantly, these effects were seen even after weeks of treatment, which aligns with the HF benefits observed in trials such as the DEFINE-HF trial [95]. These results underscore the relevance of intraglomerular hemodynamic changes and their role in SGLT2i-induced HF benefits. Another clinically relevant mechanism that has gained increasing attention is the effect of SGLT2is on erythropoiesis. Clinical trials have demonstrated increases in hematocrit following SGLT2i therapy as a result of induced erythropoiesis. These findings suggest restoration of renal erythropoietin production and improved oxygen-delivering capacity [96]. Enhanced erythropoiesis may therefore contribute to improved tissue oxygenation and represent another potential mechanism underlying the cardiovascular and renal benefits observed with SGLT2 inhibition.

### 3.6. Potential Pulmonary Protective Effects of SGLT2 Inhibitors

Although pulmonary mechanisms remain less well characterized than renal and cardiovascular effects, emerging evidence suggests SGLT2 inhibitors may influence respiratory physiology through indirect hemodynamic and anti-inflammatory pathways. Dapagliflozin’s well established cardioprotective benefits have been the centerpiece of recent research, yet a growing body of evidence suggests that its therapeutic reach may extend into other organ systems, including the lungs. Emerging studies indicate that dapagliflozin and other SGLT2 inhibitors may reduce the risk of respiratory impairment and, in certain conditions, even improve diseased pulmonary tissue. This has prompted researchers to consider whether the systemic metabolic and hemodynamic effects of SGLT2is translate into clinically meaningful pulmonary benefits. One notable study demonstrated that dapagliflozin significantly improved lung fluid volumes compared to placebo [95]. Although originally designed to evaluate heart failure outcomes, the observed reduction in pulmonary fluid burden highlights a possible role for dapagliflozin in relieving congestion at the level of the lung. These findings expand the drug’s therapeutic potential beyond its proximal tubular mechanism and suggest downstream cardiopulmonary interactions that may benefit patients with diabetes and other diseases as well. Beyond congestion relief, SGLT2is may also influence lung inflammation and fibrosis. A study using empagliflozin found that treatment reduced expression of proinflammatory cytokines TNF-α and IL-6, mediators of pulmonary fibrosis [97]. While the experiment was not performed with dapagliflozin specifically, the shared mechanism across the drug class suggests a broader anti-inflammatory and antifibrotic potential. These findings raise the possibility that SGLT2 inhibitors may eventually have therapeutic relevance in chronic inflammatory or fibrotic lung diseases beyond diabetes associated complications. Further evidence of pulmonary benefit emerges from population level studies. A comparative analysis of SGLT2is, including dapagliflozin, against dipeptidyl peptidase-4 inhibitors (DPP-4is) revealed that patients with type 2 diabetes treated with SGLT2is experienced a 40% lower risk of adverse respiratory events, along with reduced risk of pneumonia, acute pulmonary edema, and respiratory failure [98]. Such a reduction suggests a meaningful class effect that goes beyond glucose control, potentially tied to improved hemodynamics, reduced inflammation, or modulation of cardiorespiratory physiology. Another study explored the hypothesis that despite the lack of SGLT2 receptors in pulmonary tissue, the glycosuria inducing action of SGLT2is may indirectly benefit the lungs by reducing carbon dioxide retention and lowering the likelihood of pneumonia, a major driver of chronic obstructive pulmonary disease (COPD) exacerbations. In patients with T2DM and COPD, SGLT2i therapy was correlated with a 38% reduced risk of severe exacerbations compared with sulfonylureas, despite sulfonylureas being prescribed four times more often [99]. This contrast not only underscores the potential pulmonary advantages of SGLT2is but also exposes a notable gap between prescribing patterns and emerging evidence. Collectively, these studies reveal a compelling and underrecognized dimension of SGLT2 inhibitor pharmacology. While dapagliflozin was not the sole agent examined across all studies, the consistency of pulmonary benefit across the drug class spanning improved lung fluid dynamics, reduced inflammatory signaling, and decreased real world respiratory morbidity suggests that SGLT2is may hold broader therapeutic value for patients at risk of respiratory complications. Although current evidence remains limited compared with renal and cardiovascular literature, these findings support continued investigation into the pulmonary effects of SGLT2 inhibition to answer important questions about whether current diabetic treatment methods fully reflect the potential benefits of SGLT2 inhibitors.

### 3.7. Shared Mechanistic Themes Underlying the Systemic Effects of SGLT2 Inhibitors

Although the effects of SGLT2 inhibitors are often discussed within organ-specific contexts, accumulating evidence suggests that many of their protective actions converge on several interconnected biological pathways. Across renal, cardiovascular, and potentially pulmonary systems, SGLT2 inhibition consistently modulates oxidative stress, inflammatory signaling, mitochondrial function, cellular metabolism, and fibrosis-related pathways.

One of the most consistently observed effects of dapagliflozin is suppression of inflammatory signaling, particularly pathways involving NLRP3 inflammasome activation, NF-KB signaling, and proinflammatory cytokine production [100]. Because chronic inflammation contributes to tissue remodeling and progressive organ dysfunction across multiple diseases, attenuation of these pathways may provide a common mechanistic basis for the broad therapeutic benefits of SGLT2 inhibitors.

Oxidative stress and mitochondrial dysfunction also emerge as recurring mechanistic themes. By improving mitochondrial efficiency, reducing ROS production, and altering cellular substrate utilization, SGLT2 inhibitors appear to improve energetic homeostasis in metabolically stressed tissues [82]. These effects may be especially important in organs with high energy demands, including the kidney and heart.

Collectively, these findings support the concept that SGLT2 inhibitors function not only as glucose-lowering agents, but as broad regulators of cellular stress adaptation and organ protection. Understanding how these interconnected mechanisms interact across tissues may be critical for identifying future therapeutic applications of SGLT2 inhibition beyond diabetes and CKD.

## 4. Limitations and Alternative Therapies

### 4.1. Limitations

Although dapagliflozin and other SGLT2is offer significant benefits for glycemic control, renal protection, and cardiovascular health, important limitations and adverse effects must be considered when determining patient suitability. In a controlled clinical trial involving adults with type 1 diabetes, dapagliflozin effectively reduced HbA1c and body weight but was also associated with an increased risk of diabetic ketoacidosis (DKA). All cases were successfully treated, indicating that the risk is manageable with appropriate monitoring; however, DKA remains a clinically meaningful safety concern when selecting therapeutic options for this population [101]. Euglycemic DKA is typically uncommon in type 2 diabetes, yet a documented case of severe euglycemic ketoacidosis following dapagliflozin therapy in a patient with T2DM demonstrates that the complication can extend beyond the type 1 diabetic population [102]. The occurrence of ketoacidosis in both T1DM and T2DM underscores the need for careful patient selection, early recognition of symptoms, and ongoing investigation into the mechanisms and predictors of SGLT2 inhibitor-associated DKA. Another rare but serious adverse effect linked to SGLT2 inhibitor therapy is Fournier’s gangrene (FG), a life-threatening necrotizing infection of the perineum. While empagliflozin has been implicated more frequently, case reports have also described FG in patients receiving dapagliflozin, indicating that the risk may be a class effect rather than drug specific [103]. Although the absolute incidence is extremely low, awareness of this potential complication is important, particularly in patients with predisposing risk factors such as immunosuppression or recurrent genitourinary infections. Another important point to address is the impact of SGLT2is on muscle mass and function. Administration of dapagliflozin has been shown to decrease skeletal muscle mass in T2DM models [104]. While this effect is important to note, the plethora of data requires analysis beyond the scope of this manuscript. Overall, while the therapeutic benefits of dapagliflozin generally outweigh its risks, these adverse events highlight the importance of individualized treatment planning, patient education, and continued research aimed at optimizing the safety profile of SGLT2 inhibitors.

### 4.2. Alternative Treatment and Combination Therapy Options of SGLT2is

Although SGLT2 inhibitors offer extensive metabolic, renal, and cardiovascular benefits, several other antihyperglycemic drug classes serve as effective alternatives or adjuncts in the management of type 2 diabetes. Dipeptidyl peptidase-4 (DPP-4) inhibitors, for example, exert glucose-dependent glucose-lowering effects and help minimize glycemic variability. In a randomized controlled trial comparing dapagliflozin with DPP-4 inhibitors, both therapies achieved comparable reductions in glucose fluctuations [105]. Similarly, a head-to-head study evaluating gemigliptin versus dapagliflozin reported that gemigliptin produced a greater reduction in mean amplitude of glycemic excursions [106]. These findings highlight DPP-4 inhibitors as viable alternatives for patients who may not tolerate or qualify for SGLT2 inhibitor therapy. Beyond monotherapy, combination regimens incorporating SGLT2 inhibitors have gained considerable interest. For instance, co-administration of metformin or sulfonylureas with the SGLT2 inhibitor canagliflozin resulted in superior glycemic improvement and weight reduction compared with placebo [107]. Another emerging strategy includes combining SGLT2 inhibitors with glucagon-like peptide-1 receptor agonists (GLP-1 RAs). Evidence from both preclinical and clinical studies indicates that this dual approach offers additive metabolic benefits including enhancing glycemic control, reducing body weight, and potentially providing cardioprotection [108]. Studies have shown that administration of SGLT2 inhibitors with angiotensin-converting enzyme inhibitors can increase kidney failure-free survival in patients with albuminuric CKD [109]. Although this study focused on patients without diabetes, the renoprotection offered by this combination therapy may add years of life to patients struggling with CKD. Together, these alternative and adjunctive therapies broaden the therapeutic landscape for individualized T2DM management.

## 5. Conclusions

SGLT2 inhibitors have evolved from glucose-lowering agents into multifunctional therapies with broad systemic effects. While their primary mechanism involves inhibition of glucose reabsorption in the proximal tubule, extensive evidence demonstrates that their clinical benefits extend far beyond glycemic control. This review highlights the diverse mechanisms through which dapagliflozin influences renal physiology, including modulation of epithelial transport proteins, suppression of inflammatory signaling pathways, restoration of autophagy, and protection against podocyte injury. These effects collectively contribute to improved sodium handling, reduced oxidative stress, and preservation of kidney structure and function. Importantly, many of these mechanisms appear to operate independently of glucose lowering, underscoring the pleiotropic nature of SGLT2 inhibition. Although many of the mechanisms discussed are promising, it is important to note that this data warrants further investigation before definitive conclusions can be drawn. These findings are potentially beneficial for pulmonary fibrosis and diabetic populations; however, further clinical data is needed.

Beyond the kidney, dapagliflozin and other SGLT2is exert significant cardioprotective effects and modulate cardiovascular outcomes. Improvements in cardiac energetics, mitochondrial function, and inflammatory regulation provide a mechanistic basis for the consistent reductions in heart failure and cardiovascular mortality observed in clinical trials along with the slowing of chronic kidney disease progression, even in non-diabetic populations. Emerging data further suggest potential benefits in pulmonary physiology, highlighting the expanding therapeutic scope of this drug class.

Taken together, these findings support a shift in our understanding of SGLT2is from targeted metabolic therapies to system-wide modulators of cellular and organ function. The ability of dapagliflozin to simultaneously regulate multiple interconnected pathways highlights its potential as a disease-modifying agent across a range of chronic conditions.

Despite these advances, key mechanistic questions remain unresolved. The molecular triggers that link SGLT2-independent pathways across tissues, the relative contribution of glycosuria versus direct cellular actions, and the long-term impact of these effects in humans all warrant additional study [110]. As research continues to uncover new dimensions of SGLT2is biology, it becomes increasingly clear that SGLT2 inhibition represents a powerful therapeutic strategy with far-reaching novel clinical applications beyond diabetes and CKD.

In conclusion, dapagliflozin represents a new class of therapeutics defined not only by its metabolic effects but by its capacity to modulate complex biological systems. Continued investigation into these mechanisms will be essential for fully realizing the clinical potential of SGLT2is and advancing the treatment of cardiometabolic and renal diseases. Future studies integrating molecular biology, systems physiology, and long-term renal outcomes will be essential to determine whether SGLT2 inhibitors represent not only metabolic therapies, but a broader platform for multi-organ disease modification.

## Data Availability

The figures can be provided for educational purposes after being requested from the corresponding author in accordance with the Journal’s policies.

## Abbreviations

The following abbreviations are used in this manuscript:

BBM	brush border membrane
CKD	chronic kidney disease
COPD	chronic obstructive pulmonary disease
DKA	diabetic ketoacidosis
DKD	diabetic kidney disease
DPP-4	dipeptidyl peptidase-4
ELISA	enzyme linked immunosorbent assay
EMT	epithelial–mesenchymal transition
FG	Fournier’s gangrene
GLP-1	glucagon-like peptide-1 receptor
HF	heart failure
HFD	high-fat diet
KIM-1	kidney injury molecule 1
MDA	malondialdehyde
NAG	N-acetyl-β-D-glucosaminidase
NHE3	Na^+^/H^+^ exchanger
NGAL	neutrophil gelatinase-associated lipocalin
NKA	Na^+^/K^+^ ATPase
ROS	Reactive oxygen species
SGLT2i’s	Sodium glucose cotransporter-2 inhibitors
T2DM	type 2 diabetes mellitus
TGF	tubuloglomerular feedback
UUO	unilateral ureteral obstruction
